# CBCT assessment of maxillary sinus dimensions in a Saudi subpopulation: a retrospective study

**DOI:** 10.1080/07853890.2026.2617721

**Published:** 2026-01-21

**Authors:** Abdullah F. Alshammari, Ahmed A. Madfa

**Affiliations:** ^a^ Department of Basic Dental and Medical Science, College of Dentistry, University of Ha’il, Ha’il, Kingdom of Saudi Arabia; ^b^ Medical and Diagnostic Research Center, University of Ha’il, Ha’il, Saudi Arabia; ^c^ Department of Restorative Dental Science, College of Dentistry, University of Ha’il, Ha’il, Kingdom of Saudi Arabia

**Keywords:** Asymmetry, cone-beam computed tomography (CBCT), Ha’il region, maxillary sinus, morphometry, Saudi population, sexual dimorphism

## Abstract

**Background:**

The maxillary sinus (MS) exhibits interindividual anatomical variation influenced by sex, age, and craniofacial morphology. Understanding these variations is essential for surgical, endodontic, and implant planning. However, morphometric data for Saudi subpopulations, particularly from northern regions such as Ha’il, remain limited.

**Objective:**

To assess MS morphometric dimensions using cone-beam computed tomography (CBCT) in a Saudi subpopulation from the Ha’il region and to evaluate variations according to sex, age, and laterality.

**Methods:**

A retrospective CBCT-based cross-sectional study analysed 1,018 scans of Saudi adults aged 18–70 years. Sinus width, length, area, and perimeter were measured bilaterally on standardised coronal sections. Statistical analyses included Mann–Whitney U, Wilcoxon signed-rank, Kruskal–Wallis, and repeated-measures ANCOVA tests, with significance set at *p* < 0.05.

**Results:**

Males demonstrated significantly larger MS dimensions than females, particularly for area (*p* = 0.043), perimeter (*p* = 0.045), and width after covariate adjustment (*p* = 0.007). No significant age-related differences were observed across adult groups. Although right–left differences were statistically significant for all parameters (*p* < 0.01), the magnitude of asymmetry was minimal and clinically insignificant. Overall, MS dimensions remained stable throughout adulthood with a slight right-sided laterality predominance.

**Conclusion:**

CBCT-based morphometric assessment of the MS in a Saudi subpopulation from the Ha’il region revealed significant sex-related differences, minimal laterality-related asymmetry, and stable dimensions across adult age groups. These region-specific normative data enhance anatomical understanding and support improved diagnostic accuracy and procedural safety in implantology, oral surgery, maxillofacial practice, and forensic applications.

## Introduction

The maxillary sinus (MS) is the largest of the paranasal sinuses and the first to develop during intrauterine life. It is a paired, air-filled cavity within the maxilla, lined by the Schneiderian membrane, which extends from the floor of the orbit superiorly to the alveolar process inferiorly [1]. Its size, shape, and internal structure show significant interindividual variation, which is impacted by functional, developmental, genetic, and environmental factors [2,3]. Age, sex, craniofacial pattern, and the degree of pneumatization have been associated with morphological variations [4,5].

In many therapeutic specialties, a thorough understanding of the dimensions and variability of the MS is essential. Planning sinus floor elevation and preventing complications such as Schneiderian membrane perforation, oroantral communication, and implant migration into the sinus cavity require precise knowledge of sinus anatomy in implant dentistry [6,7]. In endodontics, the proximity of the posterior maxillary roots to the sinus floor must be carefully evaluated before surgery to avoid sinus involvement during periapical or surgical operations [8,9]. Furthermore, understanding sinus morphology facilitates diagnosis, surgical access, and postoperative care in otolaryngology, orthodontics, and oral and maxillofacial surgery [10].

Traditionally, two-dimensional (2D) imaging modalities such as panoramic and cephalometric radiographs have been used to examine the MS. However, these techniques have certain drawbacks, such as image distortion, mistakes in magnification, and the superimposition of anatomical elements [11]. Many of these issues have been overcome with the introduction of cone-beam computed tomography (CBCT), which produces high-resolution three-dimensional (3D) images [12]. CBCT enables detailed analysis of sinus anatomy, allowing clinicians to assess linear, volumetric, and morphological characteristics with precision [13,14].

In sinus augmentation procedures, CBCT plays a critical role by enabling accurate three-dimensional assessment of maxillary sinus anatomy, including sinus width, residual alveolar bone height, Schneiderian membrane thickness, and anatomical variations such as septa. This detailed evaluation enhances surgical planning and has been shown to reduce intraoperative complications during sinus floor elevation and implant placement [15].

Numerous CBCT-based studies have examined the size and shape of the maxillary sinus in different populations, revealing notable variations according to bone type, sex, and ethnicity [16–19]. These findings emphasise the importance of population-specific morphometric databases for accurate diagnosis and individualised treatment planning. However, thorough morphometric data in the Saudi Arabian setting remain scarce. Few studies have explored the specific anatomical and dimensional features of the maxillary sinus in Saudi individuals, despite the growing use of CBCT in clinical practice for implantology, sinus floor augmentation, and maxillofacial assessment.

Most Saudi studies have focused on isolated parameters, such as sinus volume or the prevalence of septa, without providing a comprehensive assessment of sinus length, width, area, and perimeter in relation to demographic variables such as sex, age, and laterality [20–22]. Moreover, limited data are available from northern regions, as previous studies have mostly been conducted in major cities such as Riyadh, Jeddah, and Dammam. To date, no studies have used CBCT to assess the maxillary sinus dimensions in individuals from Saudi Arabia’s Ha’il area. Given the country’s diverse ethnic, genetic, and environmental backgrounds, regional differences may affect sinus morphology and craniofacial characteristics. This lack of region-specific morphometric data from Ha’il represents a critical gap in the current anatomical and radiological understanding of the Saudi population. Addressing this gap is essential to establish reliable normative data and improve clinical decision-making in sinus grafting, implant placement, and oral surgery.

Given the limited availability of CBCT-based morphometric studies from northern Saudi Arabia, particularly the Ha’il region, there is a clear need for population- and region-specific anatomical data. Therefore, this retrospective study assessed MS dimensions in a Saudi subpopulation from the Ha’il region using CBCT. The study also investigated potential differences according to age, sex, and laterality (left or right). This study aims to generate region-specific morphometric data that may support clinicians and researchers involved in maxillofacial diagnosis and treatment planning. The null hypothesis was that MS dimensions in individuals from the Ha’il region do not differ significantly according to their age, sex, or laterality.

## Methods

This study was conducted in accordance with the ethical principles of the Declaration of Helsinki. Ethical approval was obtained from the Institutional Review Board of the University of Ha’il (approval No. H-2025-609). The Ethics Committee of the University of Ha’il College of Dentistry waived the requirement for individual informed consent because of the study’s retroactive design. All patient data were anonymised before analysis to ensure privacy and confidentiality.

### Study design and setting

This retrospective, cross-sectional observational study used CBCT scans to assess the anatomical characteristics of the MS in a Saudi subpopulation. The study was conducted at a major dental imaging centre in the Ha’il region, Saudi Arabia, between 1 January 2024 and 1 January 2025.

Saudi patients were included if they met the following criteria: (1) age ≥18 years; (2) presence of a fully developed permanent dentition, with the exception of third molars; and (3) absence of missing maxillary posterior teeth (premolars and first and second molars). The presence, absence, or impaction status of third molars was not considered an exclusion criterion, as third molars do not contribute to the maxillary sinus morphology evaluated in this study.

Eligible CBCT scans were retrospectively selected from patient records and had been acquired for various dental indications, including implant planning, orthognathic assessment, evaluation of impacted third molars, and investigation of cystic or tumour lesions not involving the maxillary sinus.

Patients were excluded if CBCT images demonstrated pathological or traumatic conditions directly affecting the maxillary sinus, including fractures, inflammatory sinus disease, cysts, tumours, residual roots, or previous sinus surgery. Additional exclusion criteria included metabolic bone disorders, craniofacial asymmetry, congenital or syndromic conditions, ongoing orthodontic treatment, poor image quality due to motion artefacts or metallic scatter, and an insufficient field of view preventing accurate sinus delineation.

### Sample size calculation

The minimum required sample size was calculated as 411, based on a 95% confidence level (CI), a 5% margin of error (*E*), and an expected proportion (*P*) of 50% [18]. A total of 1,018 CBCT scans were included to enhance generalisability and reliability.

### Data collection and image acquisition

A Carestream CS 8100 3D device (Carestream Dental LLC, Atlanta, GA, USA) was used to acquire CBCT images. The operating parameters of the X-ray generator were 60–90 kV, 2–15 mA, and 140 kHz. The device allows volumetric reconstruction and uses a CMOS flat-panel detector. The minimum voxel size was 75 µm, the FOVs were 4 × 4 cm, 5 × 5 cm, 8 × 5 cm, or 8 × 8 cm, and the exposure times ranged from 3 to 15 s. CS 3D Imaging Software (Carestream Dent LLC, Atlanta, USA) was used for image analysis.

Of approximately 32,400 reviewed scans, 1,018 met the inclusion criteria. To ensure methodological accuracy and consistency, the primary examiner underwent systematic calibration and training before data collection. Two qualified examiners (AFA and AAM) with more than ten years of clinical and radiological experience performed the measurements.

The researchers collaboratively reviewed 20 randomly selected CBCT scans to establish anatomical landmarks, measurement criteria, and image orientations for a standardised measurement protocol. To verify adherence to this approach, the first 50 cases were jointly reviewed. Ten per cent of the analysed cases were then subjected to random quality control checks. Interobserver reliability was assessed using a randomly selected subset comprising 25% of the scans, which were independently remeasured by the senior observers. The results demonstrated excellent measurement reliability, with an intraclass correlation coefficient (ICC) greater than 0.89 and Cohen’s κ of 0.94.

Before image analysis, each patient’s demographic information, including age and sex, was recorded. Coronal CBCT slices were used for all maxillary sinus measurements, as they provide the most accurate view of the sinus’s mediolateral and anteroposterior extents. All CBCT images were analysed using DICOM-based multiplanar reconstruction, allowing dynamic navigation across axial, coronal, and sagittal planes to ensure correct image orientation and standardised positioning prior to measurement. Sinus dimensions were obtained only after confirming appropriate alignment of anatomical landmarks through interactive DICOM navigation, in accordance with established CBCT analysis protocols.

The patient’s head attitude was standardised such that the Frankfort horizontal plane was parallel to the floor before measurement, ensuring reproducibility and uniformity across scans. A single coronal slice was selected for each CBCT scan at the level of the maxillary sinus ostium, representing the region with the largest anteroposterior and mediolateral dimensions. This standardised slice minimised variability due to anatomical asymmetry or slice selection and served as the reference for all subsequent dimensional and planimetric measurements.

Four primary parameters were evaluated to describe the morphometry of the maxillary sinus:

1. Sinus width (mm): The maximum transverse dimension of the sinus cavity, defined as the linear distance between the most lateral point of the lateral sinus wall and the corresponding medial boundary of the sinus chamber.
2. Sinus length (mm): The greatest anteroposterior distance of the sinus cavity, measured from the most anterior to the most posterior point along the sinus wall, to determine the depth and extent of the sinuses.
3. Sinus area (cm2): Using CS 3D Imaging Software (Carestream Dent LLC, Atlanta, GA, USA), the whole cross-sectional area of the sinus cavity was obtained by manually tracing the sinus border. The software automatically calculated the enclosed area, ensuring precise and consistent planimetric evaluation.
4. Sinus perimeter (cm): The program simultaneously generated the length of the traced sinus border, representing the overall contour of the sinus in the selected coronal slice.

**Figure 1.**
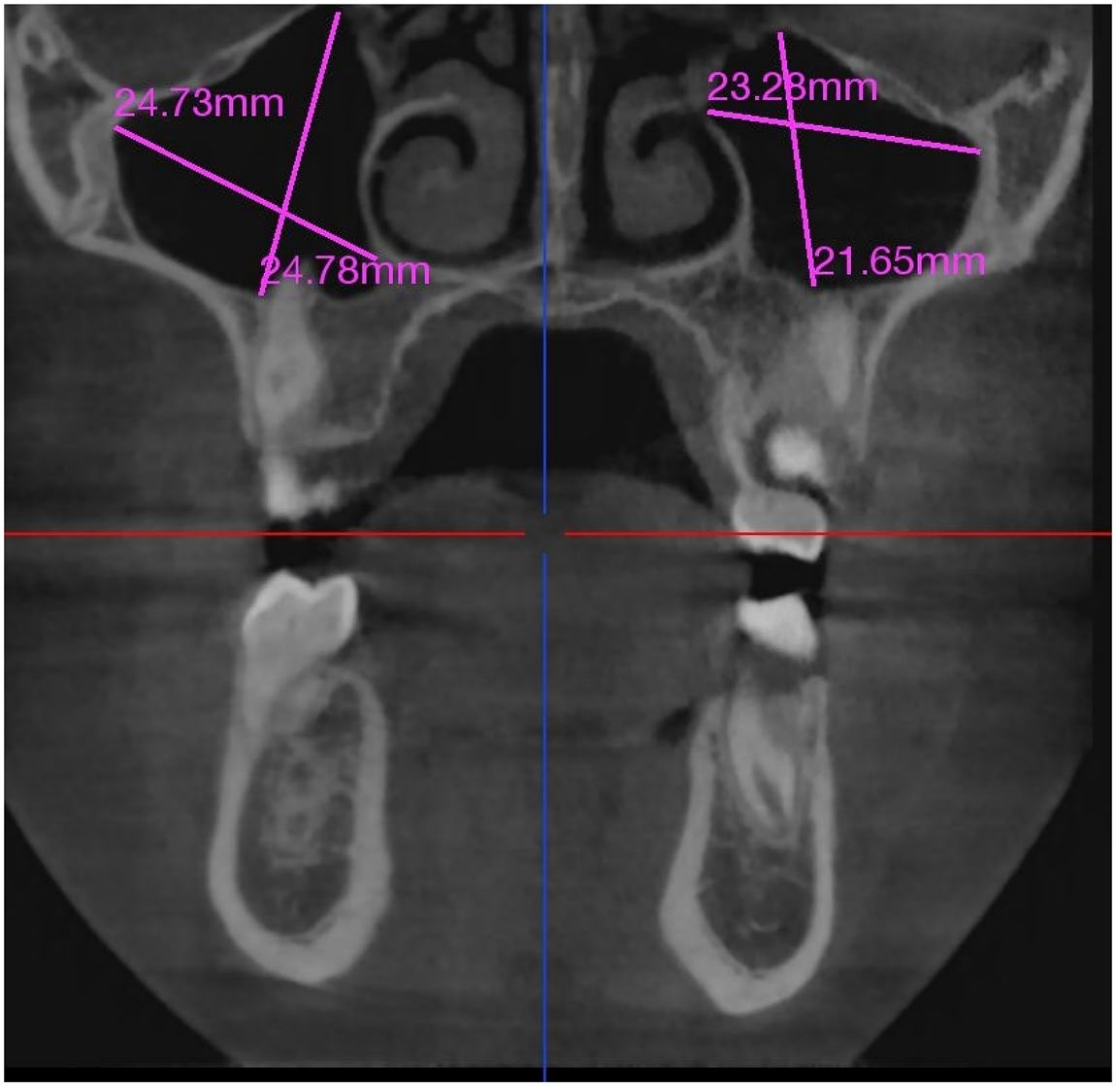
Coronal CBCT view demonstrating linear measurements of the maxillary sinuses.

All measurements were taken bilaterally, allowing comparison of each patient’s right and left maxillary sinuses directly (Figure 1). Anatomical landmarks were carefully considered during each measurement to ensure accuracy and repeatability.

### Statistical analysis

All statistical analyses were conducted using IBM SPSS Statistics software, version 23.0 (IBM Corp., Armonk, NY, USA). The Shapiro–Wilk test was used to determine the normality of the data distribution before inferential analysis. Non-parametric statistical techniques were used for the subsequent analyses, as the findings showed that the data did not follow a normal distribution. To provide a comprehensive overview of the sample’s features and sinus dimensions, descriptive statistics were computed for each measured parameter. The Mann–Whitney *U* test was used to compare the dimensions of the maxillary sinuses in male and female participants and identify significant sex-related differences. The Kruskal–Wallis test was used to assess differences in sinus measurements among age groups. Additionally, the Wilcoxon signed-rank test was used to evaluate any asymmetry in paired comparisons between the right and left maxillary sinuses among the same individuals. Furthermore, a repeated-measures analysis of covariance (ANCOVA) was performed to account for the impact of confounding factors. Age and sex were included as factors to account for their effects, and within-subject differences between the right and left sinus dimensions were assessed. For all statistical tests, a significance threshold of *p* < 0.05 was applied.

## Results

### Sample characteristics

A total of 1,018 CBCT scans from Saudi adults aged 18–70 years met the inclusion criteria and were analysed to assess MS morphology. All included scans showed intact sinus walls and well-defined bony boundaries, allowing accurate and consistent measurement of the sinus width, length, area, and perimeter with respect to laterality.

The study sample had an almost equal sex distribution, comprising 516 men (50.7%) and 502 women (49.3%). To examine developmental and possible age-related variations, participants were grouped into six age categories: 18–25, 26–30, 31–40, 41–50, 51–60, and >60 years. This grouping ensured that both early and late adulthood were represented in the analysis.

### Comparison of maxillary sinus dimensions by sex

As summarised in Table 1, men consistently showed higher mean values than women across nearly all sinus parameters across both laterality categories, suggesting a general pattern of sexual dimorphism in sinus morphology.

**Table 1.** Comparison of maxillary sinus dimensions (width, length, area, perimeter) by gender, age group, and laterality (right and left).

Maxillary sinus	Sex (mean rank)			Age groups (mean rank)						
	Male	Female	*p* value	18–25 years old	26–30 years old	31–40 years old	41–50 years old	51–60 years old	>60 years old	*p* value
**Right**										
** Width**	524.75	497.61	.144	499.37	498.38	543.33	480.98	516.70	511.49	.271
** Length**	527.69	495.32	.081	522.74	493.07	535.92	467.92	521.11	488.27	.154
** Area**	529.09	491.54	.043*	509.70	491.67	542.92	469.08	514.14	500.88	.137
** Perimeter**	530.46	493.16	.045*	511.35	491.81	544.12	471.63	516.10	500.01	.148
**Left**										
** Width**	498.24	518.28	.281	521.42	509.79	522.53	495.98	484.80	481.71	.767
** Length**	521.95	498.88	.214	523.21	504.32	540.59	463.94	504.13	477.96	.083
** Area**	508.27	509.57	.944	523.59	507.18	531.41	477.83	491.93	484.63	.360
** Perimeter**	509.17	508.86	.987	524.33	506.32	532.52	476.23	491.81	483.78	.305

*p* value calculated using Mann–Whitney test. (*) means statistically significance for age (*p* value <0.05).

*p* value calculated using Kruskal–Wallis test. (*) means statistically significance for age (*p* value <0.05).

For the right maxillary sinus, significant differences were observed between males and females in both area (*p* = 0.043) and perimeter (*p* = 0.045), indicating that men tend to have a slightly larger sinus cavity and contour length. However, differences in width and length were not significant (*p* > 0.05).

In the left sinus, although males also showed higher mean ranks, the differences did not reach statistical significance for any parameter (*p* > 0.05). Overall, the data suggest that sexual dimorphism is more pronounced on the right-sided laterality of the maxilla. This observation may reflect the generally larger craniofacial dimensions and stronger musculature commonly observed in males.

### Age-related variation

When sinus dimensions were compared across the six age groups, no significant differences were found with respect to laterality (all *p* > 0.05). Minor fluctuations were observed, most notably a slight increase in mean ranks in the 31–40-year group, but no consistent age-related pattern was evident.

These findings suggest that once the maxillary sinus reaches full pneumatization in early adulthood, its dimensions remain stable throughout life. Even with ageing, no significant remodelling or resorption was evident in the coronal CBCT images. This stability supports the view that sinus size is largely maintained after skeletal maturity.

### Bilateral comparison and symmetry analysis

To assess possible asymmetry, the Wilcoxon signed-rank test was applied to compare the right and left sinuses (Table 2). The results showed statistically significant laterality-related differences across all measured parameters:

**Table 2.** Correlation between right and left maxillary sinus anatomical dimensions and findings.

Parameter	Negative ranks (*n*)	Positive ranks (*n*)	Ties (*n*)	*Z* value	*p* value
Width (left–right)	525	491	2	–3.073	0.002
Length (left–right)	617	395	5	–7.493	<0.001
Area (left–right)	592	422	0	–6.213	<0.001
Perimeter (left–right)	606	410	1	–6.433	<0.001

*p* value calculated using Wilcoxon signed ranks test. (*) means statistically significance (*p* value <0.05).

Width: *Z* = –3.073, *p* = 0.002
Length: Z = –7.493, *p* < 0.001
Area: *Z* = –6.213, *p* < 0.001
Perimeter: *Z* = –6.433, *p* < 0.001

**Figure 2.**
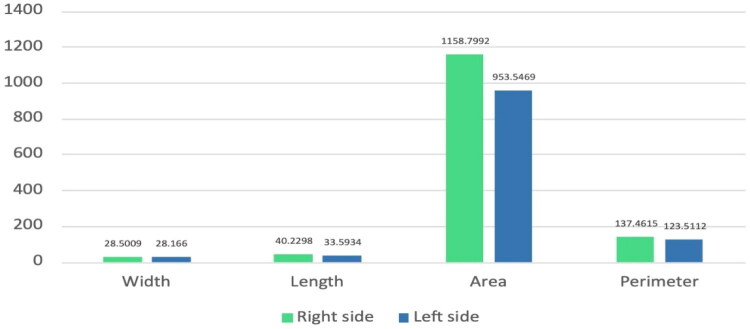
Mean maxillary sinus dimensions (width, length, area, and perimeter) between the right and left laterality as measured using CBCT imaging.

Although these differences reached statistical significance, their magnitude was small, reflecting minor physiological variation rather than clinically relevant asymmetry. Visual inspection of the plotted data (Figure 2) showed a slight tendency towards larger right-sided laterality measurements, but the substantial overlap between quartiles indicated near symmetry overall.

This subtle right-sided laterality predominance may be related to mild developmental or functional asymmetries in facial growth or masticatory dynamics; however, the overall bilateral harmony indicates that sinus dimensions are largely symmetrical in most individuals.

### Influence of age and gender: multivariate and covariate-adjusted findings

To examine the combined impact of sex and age while accounting for intra-individual correlations related to laterality (right and left), a three-way repeated-measures ANCOVA was performed (Table 3).

**Table 3.** Repeated-measures 3-way ANCOVAs of maxillary sinus dimensions (length, width, area, perimeter) controlling for age and gender.

A. Multivariate tests						
Measurement	Effect	Pillai’s trace	*F*	*df* (hypothesis)	*df* (error)	Sig.
Length	Factor1 × age	0.000	1.290	1	1014	0.593
	Factor1 × gender	0.000	0.008	1	1014	0.931
Width	Factor1 × age	0.001	1.290	1	1015	0.256
	Factor1 × gender	0.007	7.216	1	1015	0.007
Area	Factor1 × age	0.000	0.338	1	1011	0.561
	Factor1 × gender	0.000	0.063	1	1011	0.802
Perimeter	Factor1 × age	0.000	0.316	1	1014	0.574
	Factor1 × gender	0.000	0.023	1	1014	0.878
B. Within-subjects effects						
Measurement	Source	Type III SS	*df*	Mean square	*F*	Sig.
Length	Factor1 × age	2639.283	1	2639.283	0.286	0.593
	Factor1 × gender	69.632	1	69.632	0.008	0.931
Width	Factor1 × age	7.273	1	7.273	1.290	0.256
	Factor1 × gender	40.679	1	40.679	7.216	0.007
Area	Factor1 × age	2654017.763	1	2654017.763	0.338	0.561
	Factor1 × gender	494794.954	1	494794.954	0.063	0.802
Perimeter	Factor1 × age	11674.146	1	11674.146	0.316	0.574
	Factor1 × gender	863.881	1	863.881	0.023	0.878
C. Between-subjects effects						
Length	Intercept	469777.627	1	469777.627	51.169	0.000
	Age	1895.885	1	1895.885	0.207	0.650
	Gender	264.782	1	264.782	0.029	0.865
Width	Intercept	313435.570	1	313435.570	11554.1	0.000
	Age	20.992	1	20.992	0.774	0.379
	Gender	1.705	1	1.705	0.063	0.802
Area	Intercept	385886073.47	1	385886073.5	49.039	0.000
	Age	1418873.240	1	1418873.240	0.180	0.671
	Gender	705105.840	1	705105.840	0.090	0.765
Perimeter	Intercept	6195080.613	1	6195080.613	167.598	0.000
	Age	6116.672	1	6116.672	0.165	0.684
	Gender	1246.946	1	1246.946	0.034	0.854

The multivariate analysis showed that sex significantly affected sinus width (*F* = 7.216, *p* = 0.007), indicating that males tend to have wider sinuses even after adjusting for age. However, no gender-related differences were observed for sinus length, area, or perimeter (*p* > 0.05). Likewise, age did not have a significant effect on any parameter (*p* > 0.25).

No interaction effects were observed between laterality and age or between laterality and sex, suggesting that demographic variables do not influence the pattern of bilateral similarity. The between-subjects analysis showed highly significant intercepts for all parameters (*p* < 0.001), indicating natural variability within the population but no major effects of age (*p* = 0.379–0.684) or sex (*p* = 0.765–0.865) after adjustment for covariates.

## Discussion

Considering variations by sex, age, and laterality, this CBCT-based retrospective study assessed the morphometric features of the MS in a Saudi subpopulation from the Ha’il region. In terms of sinus width, area, and perimeter, the results showed a significant sex difference, with males having higher mean values than females. No age-related differences were observed across the adult age range; however, a slight but statistically significant asymmetry was found between the right and left sinuses. This CBCT-based retrospective study evaluated the morphometric characteristics of the maxillary sinus in a Saudi subpopulation from the Ha’il region, revealing sex-related differences, minimal laterality-related asymmetry, and stable sinus dimensions across adult age groups. Although retrospective in design, the primary contribution of this study lies in its region-specific analysis of an underrepresented Saudi subpopulation.

The most pronounced sex variations were in sinus width, with males exhibiting much larger bilateral values. This pattern aligns with previous CBCT studies from various regions of Saudi Arabia, which consistently found that males had larger sinus dimensions. These variations were primarily attributed to increased craniofacial dimensions, enhanced skeletal strength and endocrine-mediated influences on osteogenesis. For instance, one study reported mean sinus volumes of 13,595 mm³ for females and 16,517 mm³ for males [23], and similar sex-based differences were observed in populations from Riyadh and Al-Hasa [24]. Studies from other countries support this pattern: Kocak et al. [25] in Turkey and Prabhat et al. [26] in India found that males typically have larger sinus width and anteroposterior length than females. In addition, Chatra et al. [27] found that sinus area and perimeter are reliable indicators for estimating sex. Our data support these studies, demonstrating that male sinuses are typically larger due to the combined influence of masticatory stress, pneumatization patterns, and general craniofacial and skeletal factors.

Sex-related differences in craniofacial and maxillary sinus morphology have gained increasing attention in CBCT-based research, with evidence indicating that skeletal size, bone density, and hormonal regulation contribute to anatomical variation. Recent CBCT studies, including that by Estrela et al. [28] highlight the importance of sex as a biological variable in maxillofacial imaging, findings that are consistent with the sexual dimorphism observed in the present study. In contrast, the absence of age-related differences may reflect the stabilisation of maxillary sinus dimensions after skeletal maturity, with both biological factors and methodological aspects—such as the retrospective design, adult-only sample, and exclusion of sinus pathology—potentially limiting detectable age-related variation [28].

Several CBCT and CT studies in other populations align with the sex-related differences observed in this study [13,16,19]. Park et al. [13] found that males had longer and larger sinuses; these factors were linked to total craniofacial size rather than local sinus morphology. The current findings are also supported by Altwaijri et al., who reported wider sinuses in Saudi men [18]. The lack of age-related differences aligns with the results of Jun et al. [4,5] and Ariji et al., who showed that sinus pneumatization stabilises after adolescence. Findings from other CBCT studies [16,19] are consistent with the minor right–left asymmetry, indicating that small deviations are normal rather than pathological.

From a forensic perspective, the maxillary sinus is considered a robust anatomical structure that resists postmortem change and remains well preserved. The clear sex-based dimensional differences observed in this study demonstrate how MS morphometry may be used as a supplementary method of sex identification, particularly in situations in which traditional skeletal indications are not accessible. Region-specific reference data, such as the data provided for the Ha’il population in this study, enhance the accuracy of forensic identification and medicolegal assessment.

No significant variations were observed in sinus morphology across age groups, despite slight dimensional decreases after the fifth decade of life. This is consistent with studies showing that the MS stabilises in adulthood and reaches peak pneumatization by the third decade [29–31]. Some investigators have noted that bone remodelling and tooth loss cause the sinuses to gradually shrink with age [27,32,33]. However, others have reported minimal change; this observation is supported by the present data, which indicate that sinus dimensions show little degenerative remodelling over time and remain largely stable after reaching skeletal maturity. This stability supports the continued usefulness of sinus-based morphometric measures for adult clinical and forensic evaluation.

The study found that the right-sided laterality had slightly larger dimensions, indicating a modest but statistically significant asymmetry. These results align with previous CBCT-based studies by Bayrak et al. [34] and Przystańska et al. [35], which reported minor changes between the right and left due to developmental asymmetry or functional mastication patterns. Similarly, Motawei et al. [36] observed a slight asymmetry with no clinical significance. Therefore, although the MS is generally bilaterally symmetrical, minor individual variations should be considered in surgical planning, particularly for sinus lift and implant placement, to avoid membrane perforation or uneven grafting.

Advances in artificial intelligence (AI) are expected to play an increasingly important role in the analysis of CBCT images of the sino-nasal region. AI-based approaches enable automated segmentation, morphometric analysis, and identification of anatomical variations, thereby improving diagnostic accuracy and reducing interobserver variability. Future integration of CBCT datasets with validated AI models may support large-scale morphometric studies and personalised surgical planning in implantology and maxillofacial practice [37,38].

## Clinical implications

In implantology, sinus floor elevation, and posterior maxillary procedures, understanding sinus dimensions is essential. CBCT-based morphometric assessment helps prevent sinus membrane perforation, determine appropriate bone height, and optimise implant positioning [6,7,39]. Precise knowledge of the proximity of the sinuses to maxillary molar roots aids endodontists in avoiding sinus involvement during periapical disease care or apical procedures [8,9]. Clinicians can use the normative reference values presented here for risk assessment, postoperative evaluation, and procedure planning. In addition, such population-specific data support safer and more individualised treatment plans in Saudi Arabian dental and maxillofacial practice.

This study’s strengths include high interobserver reliability, exclusion of pathological confounders, and a large, balanced sample size. Compared with conventional CT imaging, CBCT provides high-resolution, three-dimensional evaluation with lower radiation exposure [11,12]. This study is limited by its retrospective design, which may introduce selection bias and restrict control over confounding factors. Additionally, the region-specific sample from the Ha’il population may limit the generalisability of the findings to other populations. The absence of volumetric analysis and exclusion of pathological cases further constrain broader clinical extrapolation, highlighting the need for future prospective, multicentre CBCT studies involving diverse populations.

## Conclusions

This CBCT-based study demonstrated clear sexual dimorphism in maxillary sinus morphology, minimal laterality-related asymmetry, and stable sinus dimensions across adult age groups in a Saudi subpopulation from the Ha’il region. These findings contribute region-specific morphometric reference data that enhance anatomical understanding and support safer, more accurate clinical decision-making in implantology, oral surgery, maxillofacial practice, and forensic applications.

## Data Availability

The datasets created and/or analysed for the current study are not publicly accessible because ethics approval was given on the grounds that only the researchers involved in the study would have access to the identified data, but they are available from the corresponding author upon justifiable request.
